# Thickness-dependent electrocaloric effect in mixed-phase Pb_0.87_Ba_0.1_ La_0.02_(Zr_0.6_Sn_0.33_Ti_0.07_)O_3_ thin films

**DOI:** 10.1098/rsta.2016.0056

**Published:** 2016-08-13

**Authors:** T. M. Correia, Q. Zhang

**Affiliations:** School of Aerospace, Transport and Manufacturing, Cranfield University, Bedfordshire MK43 0AL, UK

**Keywords:** electrocaloric, sol–gel, thin films

## Abstract

Full-perovskite Pb_0.87_Ba_0.1_La_0.02_(Zr_0.6_Sn_0.33_Ti_0.07_)O_3_ (PBLZST) thin films were fabricated by a sol–gel method. These revealed both rhombohedral and tetragonal phases, as opposed to the full-tetragonal phase previously reported in ceramics. The fractions of tetragonal and rhombohedral phases are found to be strongly dependent on film thickness. The fraction of tetragonal grains increases with increasing film thickness, as the substrate constraint throughout the film decreases with film thickness. The maximum of the dielectric constant (*ε*_m_) and the corresponding temperature (*T*_m_) are thickness-dependent and dictated by the fraction of rhombohedral and tetragonal phase, with *ε*_m_ reaching a minimum at 400 nm and *T*_m_ shifting to higher temperature with increasing thickness. With the thickness increase, the breakdown field decreases, but field-induced antiferroelectric–ferroelectric (*E*_AFE−FE_) and ferroelectric–antiferroelectric (*E*_FE−AFE_) switch fields increase. The electrocaloric effect increases with increasing film thickness.

This article is part of the themed issue ‘Taking the temperature of phase transitions in cool materials’.

## 1. Introduction

Antiferroelectric lead lanthanum zirconate stannate titanate (Pb,La)(Zr,Sn,Ti) (PLZST) compositions near the morphotropic phase boundary (MPB) have been widely considered for a wide range of applications, such as displacement transducers, pyroelectric detectors, piezoelectric actuators, etc. [1–11]. This has been due to the fact that the ferroelectric phase may be induced in these compositions by application of an electric field or stress or by varying the temperature, which brings about large strain and pyroelectric coefficient [1–7]. However, MPB PLZST compositions are characterized by large electrical hysteresis and high phase transition temperature, which may limit their uses for some applications. Several attempts have been made to overcome these limitations by introducing dopants within the PLZST lattice. In particular, the doping of PLZST with barium (Ba) has been demonstrated to reduce electrical hysteresis and bring down the paraelectric–antiferroelectric and field-induced paraelectric–ferroelectric phase transition temperatures towards room temperature [12,13]. Yet, only a few works have ever addressed the study of Ba-doped Pb_0.98_La_0.02_(Zr,Sn,Ti) bulk [12,13]. In the present work, we selected the Pb_0.87_Ba_0.1_La_0.02_(Zr_0.6_Sn_0.33_Ti_0.07_)O_3_ composition (abbreviated herein as PBLZST) in which, according to Liu *et al.* [13], the paraelectric–antiferroelectric and field-induced paraelectric–ferroelectric phase transitions occur near room temperature, a very attractive feature for many applications operating near ambient temperature.

The electrocaloric properties of thin films can be affected by extrinsic factors occurring during film fabrication; misfit stresses in epitaxial films and thermal stresses in films with large thermal expansion mismatch versus the substrate are well-known examples. Roh *et al*. have reported a thickness-dependent electrocaloric effect (ECE) in polycrystalline relaxor ferroelectric PLZT (10/65/35) films [14]. According to the authors, this observation is a result of several extrinsic factors, such as stress and dead layer formation between PLZT and the electrode, which in turn affects the dielectric, ferroelectric and electrocaloric properties of the thin film. In this paper, we aim to investigate the effect of PBLZST thin-film thickness on their crystal structure and consequent effect on dielectric, ferroelectric and electrocaloric properties.

## 2. Experimental

PBLZST thin films with different thicknesses were fabricated by a sol–gel method (see details in [15]). Four different PBLZST thin films with different thicknesses (200 nm, 400 nm, 700 nm and 830 nm) were obtained and further investigated. The crystal structure and quality of the films were characterized by X-ray diffraction (XRD). Dielectric constant measurements were carried out using a Wayne-Kerr impedance analyser at 10 kHz and *V*_AC_=0.5 V. Polarization–electric field (*P*–*E*) loops were obtained at 1 kHz by means of a Radiant Technologies RT66A ferroelectric tester, while a Peltier element was used for temperature control.

## 3. Results

**Figure 1.**
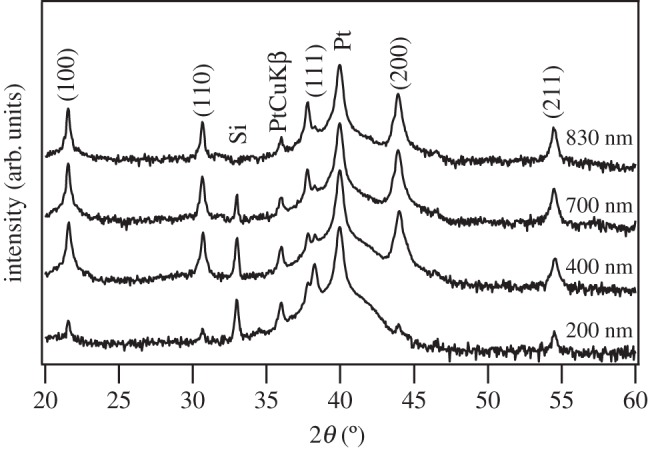
XRD patterns obtained in 200 nm, 400 nm, 700 nm and 830 nm PBLZST thin films.

**Figure 2.**
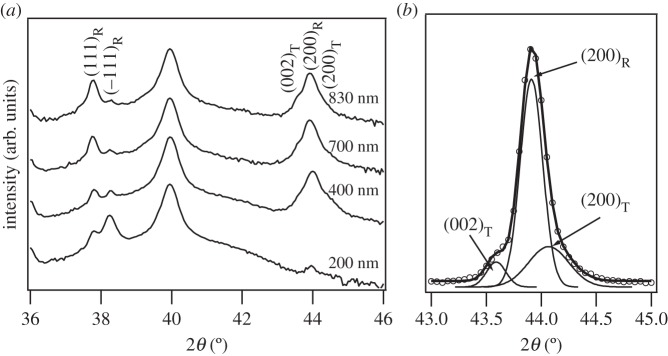
(*a*) Enlarged XRD patterns of PBLZST thin films, indicating split of pseudocubic (111) and (200) reflections. (*b*) Example of (200) peak deconvolved by three Gaussian functions.

**Figure 3.**
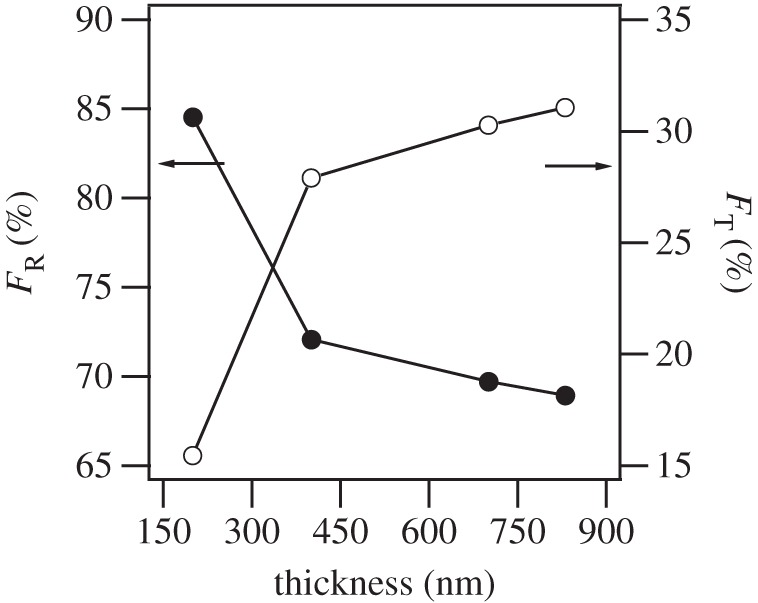
Fractions of rhombohedral (*F*_R_) and tetragonal (*F*_T_) phases as a function of PBLZST thin-film thickness.

Figure 1 shows the XRD patterns obtained in PBLZST thin films. As can be seen, the aforementioned sol–gel method [15] resulted in single-phase and pyrochlore-free thin films. The evidence of (−111)/(111) doublet peaks, as seen in the enlarged XRD spectra in figure 2*a*, also indicates that PBLZST thin films crystallize in a rhombohedral structure. Nevertheless, the asymmetrically shaped (200) reflection peak suggests the overlapping of distinct crystal plane reflections. Figure 2*b* illustrates an example of the deconvolution of the (200) peak into three Gaussian functions, ascribed to tetragonal (002)_T_ and (200)_T_ and rhombohedral (200)_R_ reflections. This outcome suggests that PBLZST thin films are characterized by a mixed rhombohedral/tetragonal phase, as opposed to the full-tetragonal phase reported in PBLZST ceramics [13]. The fractions of rhombohedral and tetragonal phases (*F*_R_ and *F*_T_, respectively) in PBLZST thin films were evaluated with recourse to the following equations:

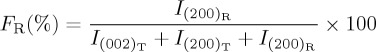
and

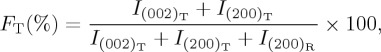
where *I*(*hkl*) is the integrated area of the reflection peak (*hkl*). As film thickness increases, the tetragonal phase is seen to gradually increase from ‘mostly’ rhombohedral film (200 nm) up to 31% tetragonal-phase film (830 nm) towards extrapolated fully tetragonal bulk PBLZST (figure 3). This may be understood by considering the fact that PBLZST thin films crystallize in a two-layered structure, as previously proposed by Kelman and co-workers [16] for PbZr_0.3_Ti_0.7_O_3_ thin films. According to this work, grains at the bottom of the film are strongly affected by misfit strain, due to lattice mismatch and difference in thermal expansion coefficients between the substrate and the film, and therefore these are compelled to undergo a tetragonal to rhombohedral transformation. On the other hand, grains at the surface are relaxed and thus remain tetragonal. As a consequence, the fraction of tetragonal grains increases with increasing film thickness, as the substrate constraint throughout the film decreases with film thickness.

**Figure 4.**
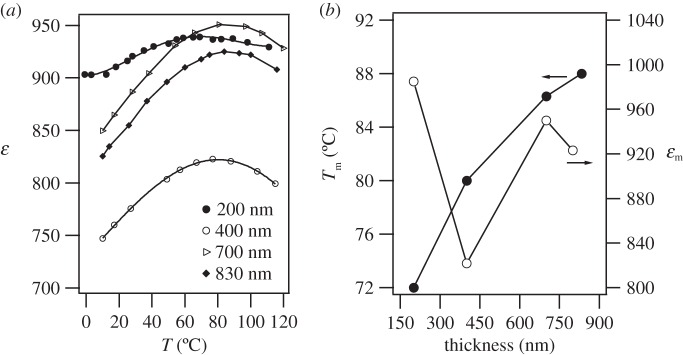
(*a*) Dielectric constant (*ε*) as a function of temperature at 1 kHz, obtained in 200 nm, 400 nm, 700 nm and 830 nm PBLZST thin films. (*b*) Maximum dielectric constant (*ε*_m_) and temperature (*T*_m_) as functions of PBLZST thin-film thickness.

Figure 4*a* illustrates the temperature dependence of the dielectric constant, *ε*, obtained in 200 nm, 400 nm, 700 nm and 830 nm PBLZST thin films. Although the paraelectric–antiferroelectric phase transition has been reported to occur at approximately 40°C in PBLZST ceramic [13], in PBLZST thin films the maximum of the dielectric constant (*ε*_m_) occurs at 70°C in 200 nm film, shifting towards higher temperatures with increasing film thickness. Moreover, *ε*_m_ reported in this work is rather lower than that previously reported in PBLZST ceramic (approx. 4200), a phenomenon that may have been brought about by a number of factors, such as smaller grain size, formation of a ‘dead layer’ between film and substrate, internal stresses and substrate clamping. If the thickness of the film further increases, it could be expected that tetragonal phase would dominate in the film and *ε*_m_ of the film would be close to that of the bulk material. Notwithstanding this fact, *ε*_m_ and the corresponding temperature (*T*_m_) are clearly film thickness-dependent, as can be seen in figure 4*b*, suggesting that both values are dictated by *F*_R_ and *F*_T_. The value of *T*_m_ shifts to higher temperature with the film thickness due to the increase of tetragonal phase, as shown in figure 3. The *ε*_m_ values do not vary monotonically with film thickness, which is suggestive that the ‘dead layer’ does not contribute significantly to PBLZST thin-film dielectric response. It is also clear that a minimum in *ε*_m_ is observed at 400 nm, which coincides with the ‘mostly’ rhombohedral to rhombohedral/tetragonal phase transition, an observation that corroborates the results reported by Pertsev *et al.* [17] and Kelman *et al.* [16].

**Figure 5.**
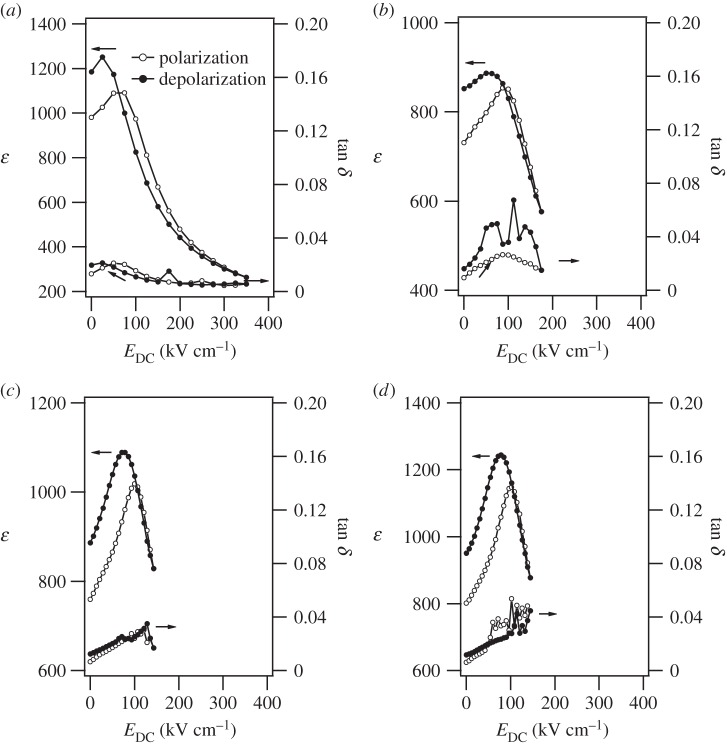
Dielectric constant (*ε*) and loss (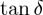) as a function of DC field (*E*_DC_) measured at 10 kHz in (*a*) 200 nm, (*b*) 400 nm, (*c*) 700 nm and (*d*) 830 nm PBLZST thin films.

**Figure 6.**
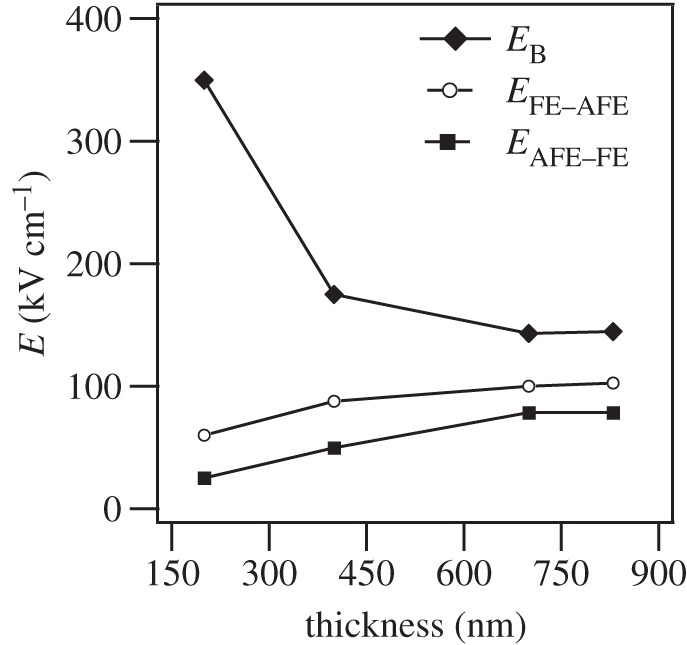
Breakdown field (*E*_B_), AFE–FE switch field (*E*_AFE−FE_) and FE–AFE switch field (*E*_*FE*−*AFE*_) as a function of PBLZST thin-film thickness.

According to the PBLZST ceramic phase diagram, the application of an electric field of 12.5 kV cm^−1^ induces an antiferroelectric–ferroelectric phase transition [13]. We have previously demonstrated that, in 200 nm PBLZST thin film, such an antipolar–polar phase transition is obtained upon application of a bias field as low as 60 kV cm^−1^, thereby resulting in a high tunability value [15]. In this paper, we investigated the impact of film thickness on the antiferroelectric–ferroelectric switch field in the fabricated PBLZST thin films. As shown in figure 5*a*–*d*, *ε* and 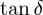 increase at room temperature with bias field *E*_DC_ up to a certain value of bias field, after which they decrease rapidly, thus suggesting a field-induced antiferroelectric–ferroelectric phase transition. Nevertheless, the antiferroelectric–ferroelectric switch field is found to shift towards higher *E*_DC_ upon increasing the film thickness. Figure 6 shows the breakdown field (*E*_B_) and the field-induced antiferroelectric–ferroelectric (*E*_AFE−FE_) and ferroelectric–antiferroelectric (*E*_FE−AFE_) switch fields as a function of film thickness: *E*_B_ decreases and *E*_AFE−FE_ and *E*_*FE*−*AFE*_ increase with increasing film thickness, respectively. Note that a great variation of breakdown and switch fields is detected at 400 nm, at which the ‘mostly’ rhombohedral to mixed rhombohedral/tetragonal phase transition occurs, as mentioned above.

**Figure 7.**
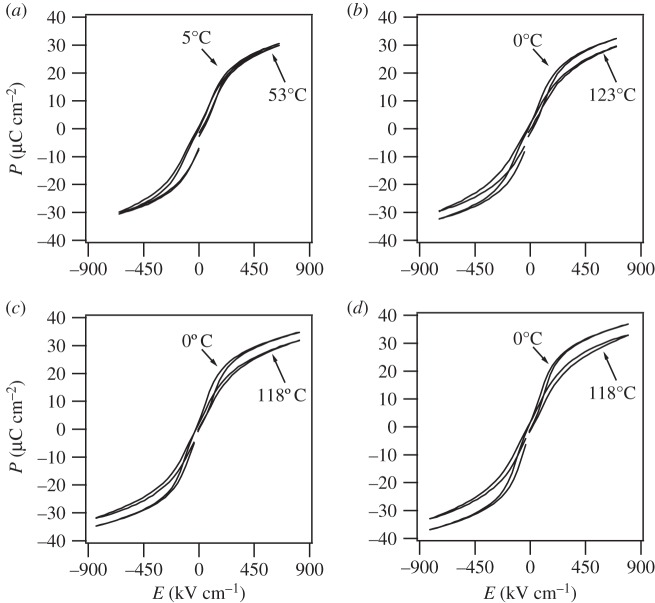
*P*–*E* loops obtained in (*a*) 200 nm, (*b*) 400 nm, (*c*) 700 nm and (*d*) 830 nm PBLZST thin films at selected temperatures and at 1 kHz. Data refer to field-cooling (FC) approach.

**Figure 8.**
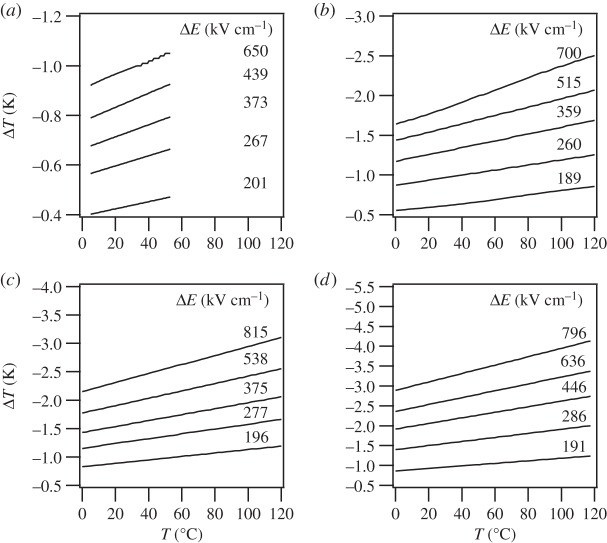
Electrocaloric temperature change (Δ*T*) as a function of temperature (*T*), measured indirectly in (*a*) 200 nm, (*b*) 400 nm, (*c*) 700 nm and (*d*) 830 nm PBLZST thin films and for different Δ*E*.

**Figure 9.**
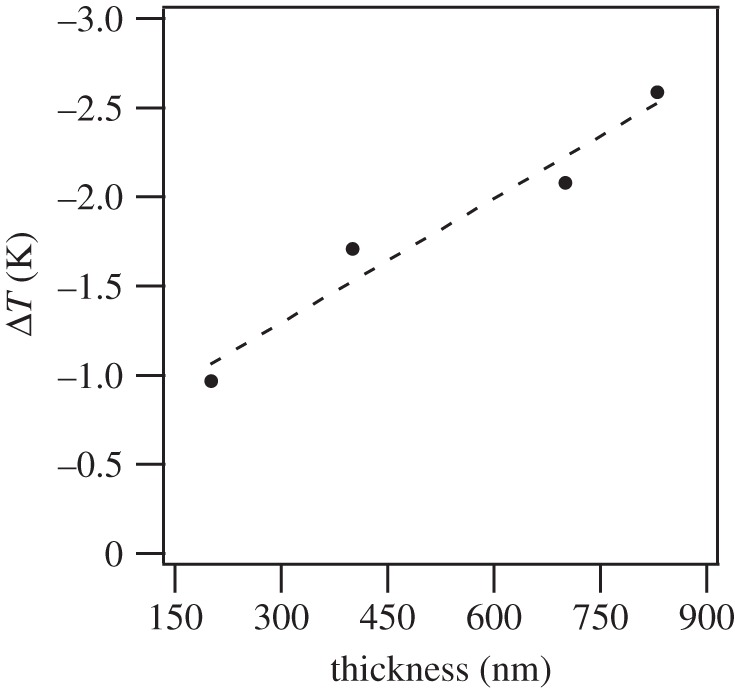
Electrocaloric temperature change (Δ*T*) as a function of thickness, at 650 kV cm^−1^ at room temperature. Data obtained from interpolation of Δ*T*(*E*).

Figure 7*a*–*d* depicts *P*–*E* loops obtained in PBLZST thin films. Low electrical hysteresis and ferroelectric-like loops characterize the studied films, a fact attributed to a low antiferroelectric–ferroelectric switch field. At temperatures above 53°C, *P*–*E* loops exhibited a leaky performance, and for this reason, ECE in the 200 nm PBLZST was measured indirectly within the 0–53°C temperature span, as opposed to 0–120°C in the thicker films. ECE, i.e. temperature change (Δ*T*) driven by the application/withdrawal of an electric field, was indirectly measured from *P*–*E* loops undertaken at different temperatures and considering the following equation:

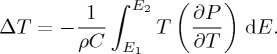
The lower integration limit was set as *E*_1_=0 due to low electrical hysteresis. Heat capacity, *C*, and density, *ρ*, were assumed to be 330 J kg^−1^ K^−1^ and 8.3 g cm^−3^, respectively. The electrocaloric Δ*T* indirectly measured on the investigated PBLZST thin films is depicted in figure 8*a*–*d*: Δ*T* is found to increase linearly with temperature, *T*. No phase transition occurs within the studied temperature range, at which a peak on the Δ*T*–*T* curve should be expected. This is not in agreement with the PBLZST ceramics phase diagram, which indicates that a field-induced paraelectric–ferroelectric phase transition is observed between 40 and 50°C for 0<*E*<30 kV m^−1^, to which the large *E* applied to the studied thin films and the effect of substrate constraint may have contributed. Furthermore, figure 9 shows that ECE increases with increasing film thickness. This may be explained by considering that, by increasing the film thickness, the effect of the substrate constraint throughout the film diminishes. This corroborates the findings of previous works reporting that ECE is, in fact, strongly affected by a misfit-strain effect, which can, in turn, be tailored by adjusting the film thickness [18–21].

## 4. Conclusion

Full-perovskite PBLZST thin films were fabricated by a sol–gel method. These films were characterized by tetragonal and rhombohedral phases, as opposed to the tetragonal phase observed in PBLZST ceramic. The fraction of tetragonal and rhombohedral grains was found to be thickness-dependent, which was duly explained within the framework of the ‘two-layered model’, according to which a rhombohedral layer develops at the bottom of the film. This is caused by the strong effect of misfit strain between the film and the substrate and the strain-free tetragonal PBLZST grains located at the film surface. The two-layered structure of PBLZST thin films resulted in film dependence of the paraelectric–antiferroelectric phase transition temperature, antiferroelectric–ferroelectric switch field and ECE.
